# DNA Barcoding of Catfish: Species Authentication and Phylogenetic Assessment

**DOI:** 10.1371/journal.pone.0017812

**Published:** 2011-03-15

**Authors:** Li Lian Wong, Eric Peatman, Jianguo Lu, Huseyin Kucuktas, Shunping He, Chuanjiang Zhou, Uthairat Na-nakorn, Zhanjiang Liu

**Affiliations:** 1 Department of Fisheries and Allied Aquacultures, Aquatic Genomics Unit, Auburn University, Auburn, Alabama, United States of America; 2 Institute of Hydrobiology, Chinese Academy of Sciences, Wuhan, Hubei Province, China; 3 School of Life Science, Southwest University, Beibei, Chongqing, China; 4 Department of Aquaculture, Faculty of Fisheries, Kasetsart University, Bangkok, Thailand; Biodiversity Insitute of Ontario - University of Guelph, Canada

## Abstract

As the global market for fisheries and aquaculture products expands, mislabeling of these products has become a growing concern in the food safety arena. Molecular species identification techniques hold the potential for rapid, accurate assessment of proper labeling. Here we developed and evaluated DNA barcodes for use in differentiating United States domestic and imported catfish species. First, we sequenced 651 base-pair barcodes from the cytochrome oxidase I (COI) gene from individuals of 9 species (and an Ictalurid hybrid) of domestic and imported catfish in accordance with standard DNA barcoding protocols. These included domestic Ictalurid catfish, and representative imported species from the families of Clariidae and Pangasiidae. Alignment of individual sequences from within a given species revealed highly consistent barcodes (98% similarity on average). These alignments allowed the development and analyses of consensus barcode sequences for each species and comparison with limited sequences in public databases (GenBank and Barcode of Life Data Systems). Validation tests carried out in blinded studies and with commercially purchased catfish samples (both frozen and fresh) revealed the reliability of DNA barcoding for differentiating between these catfish species. The developed protocols and consensus barcodes are valuable resources as increasing market and governmental scrutiny is placed on catfish and other fisheries and aquaculture products labeling in the United States.

## Introduction

Catfish (Order Siluriformes) are a diverse group of fish representing more than 3,000 species, 478 genera and 36 families [1]. Ictalurid catfish represent the largest segment of the domestic aquaculture industry in the United States, generating approximately 600 million pounds of catfish per year [2]. Imports of Pangasiid, Clariid, and Ictalurid catfish to the United States from East Asia (largely Vietnam and China) have increased rapidly over the last decade and now account for up to half of catfish sales in the U.S. [3]. Import restrictions and labeling requirements have impacted the sources and species of imported catfish, but have not substantially reduced import numbers. Anecdotal and documented cases of catfish species mislabeling (either as another catfish species or as a higher value species) are widespread. New regulations currently under development by the federal government will seek to strengthen inspection of domestic and imported catfish, including verification of correct species labeling. Further development and validation of DNA barcoding techniques and consensus sequences for catfish are therefore needed to ensure accuracy in product labeling and informed consumer choices.

DNA barcoding involves the amplification and sequencing of a short universal molecular tag of approximately 650 bp from the 5′ region of the mitochondrial cytochrome oxidase I (COI) gene [4]–[5]. DNA barcoding using COI has been widely employed in various biological fields with proven ability to differentiate closely related species in studies ranging from forensic sciences [6], molecular systematics [7] to seafood products identification [8]–[11]. Importantly, community-based efforts to develop extensive DNA barcode libraries, most notably the Barcode of Life Data Systems (BOLD), has led to the adoption of DNA barcoding technology as the gold standard for species identification and has greatly expanded the power of the technique. The BOLD database provides detailed information of COI-sequenced species including the origin and current location of voucher specimens [12]. Out of almost 30,000 fish species estimated in the world, barcodes for more than 10,000 fish species are currently recorded in the BOLD database. These COI barcodes are gathered from several sources including the Fish Barcode of Life Initiative (FISH-BOL) [13]–[14] and the Marine Barcode of Life Initiative (MarBOL, http://www.marinebarcoding.org). However, for many species, BOLD barcodes are gleaned from uncurated Genbank records and require additional validation before use.

Here we describe the testing and validation of DNA barcode techniques for domestic and imported catfish including the creation of a DNA barcode database containing eight worldwide commercialized catfish as well as two wild populations of catfish species. Species involved in this study (Table 1) included channel catfish (*Ictalurus punctatus*), blue catfish (*Ictalurus furcatus*) and hybrids of channel (♀)×blue (♂) catfish; Southeast Asian walking catfish (*Clarias batrachus*), broadhead catfish (*Clarias macrocephalus*), basa (*Pangasius bocourti*), swai or sutchi (*Pangasianodon hypopthalmus*); and African sharp-toothed catfish (*Clarias gariepinus*). To complement this study for the purpose of detecting market substitution with vulnerable species, two wild catfish species from China were also included: helmet catfish (*Cranoglanis bouderius*) and long-barbel catfish (*Hemibagrus macropterus*). Our results indicate that DNA barcoding is a powerful technique, allowing accurate identification of known, blinded, and commercial samples. As the United States heightens inspection and regulation requirements for seafood products, DNA barcoding will serve as an important tool in efforts to ensure consumer safety and fair international commerce.

**Table 1:** Catfish species used in this study.

				Similarity
Species name	Common name	Sampling location	Sample	within
			size	species (%)
*Ictalurus punctatus*	Channel catfish	Auburn University, USA	18	98
*Ictalurus furcatus*	Blue catfish	Auburn University, USA	18	98
*I. punctatus x I. furcatus*	Hybrid catfish	Auburn University, USA	19	98
*Clarias batrachus*	Walking catfish	Nakhon Ratchasima Province,	17	97
		NE Thailand		
*Clarias gariepinus*	African sharp-toothed	Nakhon Ratchasima Province,	19	98
	catfish	NE Thailand		
*Clarias macrocephalus*	Bighead catfish	Faculty of Fisheries,	16	98
		Kasetsart University, Thailand		
*Pangasius bocourti*	Basa catfish	Yasothon Province, NE Thailand	22	98
*Pangasianodon hypophthalmus*	Swai or Sutchi catfish	Nakhon Ratchasima Province,	19	98
		NE Thailand		
*Cranoglanis bouderius*	Helmet catfish	(Guangxi and Guangdong), China	10	94
*Hemibagrus macropterus*	Long-barbel catfish	(Chongqing, Guangxi, Jiangxi,	15	98
		Sichuan, Hubei, Hunan, Fujian),		
		China		

## Materials and Methods

All experimental procedures involving fish were approved by the Institutional Animal Care and Use Committee of Auburn University under PRN 2008-1386.

### Sample Collections

A total of 173 individual samples representing 9 catfish species and an Ictalurid hybrid were used in this study (Table 1). All fin clips or tissue samples were preserved in 95% ethanol (1∶10 w∶ v) upon collection. Ictalurid species were obtained from resource populations of the Department of Fisheries and Allied Aquacultures at Auburn University, USA. Pangasiid and Clariid catfish finclips were obtained from the Department of Aquaculture, Faculty of Fisheries, Kasetsart University, Thailand. In addition, four different types of catfish specimens sold as catfish fillet, catfish nugget and skinless catfish, and swai fillet (swai catfish from Vietnam) were purchased from local grocery stores (Auburn, AL) and oriental markets (Atlanta, GA). Mitochondrial COI sequence data for both *Cranoglanis bouderius* and *Hemibagrus macropterus* were obtained in collaboration with the Laboratory of Fish Phylogenetics, Institute of Hydrobiology, Chinese Academy of Sciences, China.

### DNA extraction

Fin clips or muscle tissue samples were used to extract DNA from all samples. Twenty mg starting material was transferred to a 1.5 ml centrifuge tube containing digestion buffer [15] and Proteinase K at a concentration of 100 µg/ml. DNA was isolated using the Gentra Puregene Tissue Kit (QIAGEN, USA), following manufacturer's instructions. The concentration and purity of isolated DNA were estimated using an Ultrospec 1100 Pro spectrophotometer (GE Sciences, NJ, USA) as well as electrophoresis on a 1.5% agarose gel.

### PCR Amplification

In order to amplify 651 bp fragment from the 5′ end of mitochondrial COI gene, PCR reactions were conducted using primer cocktails of C_FishF1t1 and C_FishR1t1 (Table S1) [16]. The amplification reactions were performed in a total volume of 10 µl and included 1× Invitrogen Platinum Taq Buffer, 0.25 mM each of deoxynucleotide triphosphate (dNTPs), 2.0 mM MgCl_2_, 10 pmol of each primers, 100 ng of genomic DNA, and 0.5 units of Taq DNA polymerase. The reactions were conducted using a PTC-200 DNA Engine Thermal Cycler (Bio-Rad Laboratories, Inc., CA, USA) under the following conditions: an initial denaturation at 94°C for 2 min; 35 cycles of 94°C for 30 s, 52°C for 40 s and 72°C for 1 min; and concluded with a final elongation step of 72 °C for 10 min followed by a hold at 4 °C [16]. To ensure that the reactions yielded adequate amplicon sizes, PCR products were electrophoresed and visualized on 2.0% agarose gels containing ethidium bromide (10 mg/ml).

### Mitochondrial COI Region Sequencing

Amplified PCR products were subsequently cleaned by the Exo-SAP method [17]. Five µl of PCR product, 0.7 µl of Exonuclease I 10× Buffer (New England Biolabs Inc., MA, USA), 0.5 µl of Exonuclease I (New England Biolabs Inc., MA, USA), 0.5 µl of rAPid Alkaline Phosphatase (Roche Applied Science, IN, USA), and 5.3 µl of nanopure water were incubated at 37°C for 30 min before being denatured at 80°C for 20 min. The purified products were labeled using the BigDye Terminator v.3.1 Cycle Sequencing Kit (Applied Biosystems Inc., CA, USA) in a total reaction mixture of 10 µl containing 4.94 µl of nanopure water, 1.94 µl of 5× BigDye Buffer (400 mM Tris–HCl pH 9.0 and 10 mM MgCl_2_), 2 µl of 10 pmol of M13F or M13R (Table S1), 0.12 µl of BigDye Terminator (Applied Biosystems Inc., CA, USA), and 1 µl ExoSAP products. Sequence-PCR products were cleaned up using the ethanol/EDTA precipitation method and sequenced bi-directionally on an ABI 3130×l Genetic Analyzer (Applied Biosystems Inc., CA, USA). Sequence Analysis Software Version 5.2 (Applied Biosystems, Inc.) was used to generate sequence tracefiles and contiguous read lengths.

### Data Analysis

Sequences were manually assembled using Vector NTI software (Invitrogen Inc., CA, USA). Assembled contigs were end-trimmed to a homologous region using the SeqMan program (DNASTAR Inc., WI, USA). Sequences from vouchered specimens were submitted to the GenBank Barcode database with accession numbers **JF292297-JF292429**. The edited individual contigs for each species were aligned with Vector NTI to produce consensus sequences representing each species. Voucher sequences from GenBank, reference sequences from BOLD databases and consensus sequences of each species generated from this study were compared and aligned using the CLUSTALW program. The multiple sequence alignments were processed using the BOXSHADE 3.21 server (Hoffman and Baron, http://www.ch.embnet.org/software/BOX_form.html) to illustrate the homologous relationship of each species (data not shown). Reference sequence numbers (BOLD) and Accession numbers (GenBank) for voucher species which were used to construct multiple sequence alignments (for *I. punctatus*) are listed in Table S2.

Sample identification based on the sequence similarity approach was carried out using two databases; BOLD and GenBank. The highest percent pairwise identity of the consensus sequence from each species blasted (BLASTN) against NCBI were compared to the percent specimen similarity scores of the consensus sequence from each species within the BOLD-IDS (BOLD Identification System) [12]. To test the efficiency of DNA barcoding as a species identification tool, a blind sampling test was conducted, in which samples, identity unknown except to the submitting individual, were selected and sequenced.

For sequence comparisons, pairwise genetic distances were quantified based on the Kimura 2-parameter (K2P) distance model [18] using MEGA, version 5.0 [19]. A Maximum Parsimony (MP) tree using Close-Neighbor-Interchange algorithm was constructed to display a graphical view of the catfish species studied here [20]. The robustness of the MP tree was assessed by performing bootstrapping analysis with 1000 replicates, and gaps removed by complete deletion [21]. Confidence levels estimated from the analysis were assigned to each node in the tree and a consensus sequence from *H. macropterus* was used to root the tree.

## Results

The mitochondrial cytochrome oxidase I (COI) region of all samples was successfully amplified using PCR. Table 2 shows the comprehensive barcoding identification results based on GenBank and BOLD databases. Both databases revealed definitive identity matches in the range of 96%–100% for consensus sequences of five species (*Ictalurus furcatus*, *Ictalurus punctatus*, *Pangasius bocourti*, *Pangasianodon hypophthalmu*s and *Cranoglanis bouderius*) and an Ictalurid hybrid. GenBank-based identification for all species yielded an alignment E-value of 0.0. BOLD-IDS results were in agreement with GenBank results in identification of these species, yielding 100% identity, except for *I. furcatus*, *P. bocourti* and *C. bouderius*. For example, *I. furcatus* had 100% maximum identity in Genbank, whereas the percent similarity in BOLD database for this species was 99.41%. Similarly, *P. bocourti* also showed 100% maximum identity in GenBank, whereas the percent similarity for this species in BOLD database was 99.85%.

**Table 2:** Summary of identification based on each species consensus barcoded sequence using BOLD Identification System (BOLD-IDS) and BLASTN search from GenBank.

Species studied	BOLD -IDS		GenBank (BLASTN)	
	Species identification	% similarity	Species identification	% Max identity
*Ictalurus furcatus*	*Ictalurus furcatus*	99.41	*Ictalurus furcatus*	100
*Ictalurus punctatus*	*Ictalurus punctatus*	100	*Ictalurus punctatus*	100
*Hybrid* (*I.punctatus x*	*Ictalurus punctatus*	100	*Ictalurus punctatus*	100
*I. furcatus*)				
*Clarias batrachus*	**No match** *	**0**	*Clarias batrachus*	89
*Clarias gariepinus*	*Clarias gariepinus*	99.85	***Clarias batrachus*** *	**87**
*Clarias macrocephalus*	***Clarias batrachus*** *	**99.69**	***Clarias batrachus*** *	**99**
*Pangasius bocourti*	*Pangasius bocourti*	99.85	*Pangasius bocourti*	100
*Pangasius hypophthalmus*	*Pangasianodon hypophthalmus*	100	*Pangasianodon hypophthalmus*	100
*Hemibagrus macropterus*	***No match*** *	**0**	***Hemibagrus velox*** *	**87**
*Cranoglanis bouderius*	Cranoglanis *bouderius*	97.62	Cranoglanis *bouderius*	96

*Asterisk with bolded words corresponds to problematic identifications of species in the present study using either one or both of the databases. Details are further discussed in the text.

This study also highlighted, however, existing shortcomings in BOLD and GenBank databases for catfish species. GenBank failed to discriminate *Clarias gariepinus* and *Clarias macrocephalus* from *Clarias batrachus*. At the time of analysis, GenBank only had entries listed as *C. batrachus*. However, the top GenBank hit using our *C. macrocephalus* sequences was a single *C. batrachus* sequence (99% identity). Further investigation and consistent sequences from multiple positively identified *C. macrocephalus* samples led us to conclude that this GenBank *C. batrachus* sequence is mislabeled and truly represents *C. macrocephalus*. Additional *C. batrachus* sequences in GenBank appear also to be mislabeled, and are fairly distantly related to any of the *Clarias* species studied here (87–89% identity). Further identification would be needed to determine whether these sequences represent an isolated branch of *C. batrachus* or, more likely, whether they are truly from another species. Problems with Clariid identification continued in BOLD database. BOLD-IDS relies on GenBank sequences for much of its content and misidentification issues can, therefore, easily be compounded. Our *C. batrachus* sequences returned no match because the BOLD-IDS was relying on GenBank “*C. batrachus*” sequences and uses a 97% identity cutoff in declaring matches. The GenBank *C. batrachus* sequence we had determined represented *C. macrocephalus* was again used by BOLD-IDS and strongly matched our *C. macrocephalus* sequences (99.69%). BOLD-IDS does include a legitimate *C. gariepinus* barcode and we recorded 99.85% identity matches using our *C. gariepinus* samples. Further, both BOLD-IDS (species level and public data records) and GenBank database were unable to identify *H. macropterus*. No match was garnered for *H. macropterus* from BOLD-IDS, while GenBank, lacking a *H. macropterus* sequence, returned a top hit for a related species, *Hemibagrus velox* (87% identity).

From Table 3, we found that small subsamples of catfish purchased in local grocery and oriental markets were labeled correctly. All the specimens yielded coherent and perfect results (100% matches) in both databases. Interestingly, blue catfish from the USA were more commonly retailed as fresh product in oriental markets than channel catfish, likely indicating a wild-caught fish.

**Table 3:** Description of analyzed local market samples.

Species sold as	Country	Consensus identification	% Match
Catfish fillet	USA	*Ictalurus furcatus* (Blue catfish)	100
Frozen Catfish Nugget	USA	*Ictalurus punctatus* (Channel catfish)	100
Skinless catfish	USA	*Ictalurus furcatus* (Blue catfish)	100
Swai fillet	Vietnam	*Pangasianodon hypophthalmus* (Swai catfish)	100

Consensus identification is referred to species identification based on the highest percentage similarity with their corresponding match percentage from both GenBank (BLASTN) pairwise alignment and BOLD-IDS specimen similarity.

Common name of the identified species is written next to the scientific name in parentheses.

Two specimens from each of the seven species and hybrid catfish (except *C. bouderius* and *H. macropterus*) were randomly selected by a third party for a blind sample test; with the blind sampling test yielding 100% correct species identification results. This result proved that COI barcoding is an efficient tool for unknown species identification with user bias removed.

As shown in Table 4, 651 bp of COI consensus barcodes for each species were treated as discrete units to estimate the pairwise level of genetic divergence using the Kimura 2-parameter (K2P) correction model [20]. The K2P distance matrix showed a relatively high overall mean interspecific divergence of 18.3% with a standard error of 1.3%. The K2P distance between species ranged from a low 0.8% (hybrids and *I. punctatus*) to a maximum value of 22.6% (*C. macrocephalus* and Ictalurid hybrid). All the species studied displayed low levels of conspecific divergence.

**Table 4:** Estimates of Pairwise Genetic Distances between Catfish Species under Kimura 2-Parameter Model [18].

												Mean
												Conspecific
	Species	1	2	3	4	5	6	7	8	9	10	Divergence
**1**	*I. furcatus*		0.012	0.013	0.022	0.020	0.020	0.019	0.020	0.020	0.020	0.001
**2**	*I. punctatus*	0.089		0.003	0.020	0.018	0.021	0.018	0.021	0.019	0.020	0.002
**3**	Hybrid (*I. punctatus* x	0.096	**0.008** *		0.021	0.018	0.021	0.018	0.021	0.020	0.020	0.001
	I. furcatus)											
**4**	*C. batrachus*	0.224	0.205	0.209		0.017	0.016	0.020	0.021	0.020	0.020	0.002
**5**	*C. gariepinus*	0.220	0.183	0.185	0.148		0.016	0.019	0.020	0.020	0.020	0.007
**6**	*C. macrocephalus*	0.213	0.220	0.226	0.134	0.146		0.018	0.020	0.019	0.021	0.007
**7**	*P. bocourti*	0.177	0.181	0.179	0.215	0.193	0.185		0.015	0.020	0.019	0
**8**	*P. hypophthalmus*	0.177	0.187	0.185	0.221	0.201	0.201	0.116		0.019	0.017	0.003
**9**	*C. bouderius*	0.185	0.193	0.197	0.200	0.195	0.203	0.201	0.176		0.019	0.009
**10**	*H. macropterus*	0.201	0.201	0.199	0.216	0.204	0.223	0.185	0.161	0.184		0.016

Pairwise congeneric divergence was denoted by number of base substitutions per site between species (below diagonal) with their corresponding standard error (above diagonal). Complete deletion of all codon position (1st, 2nd, 3rd and noncoding) was employed in this analysis.

*Genetic distance resulting from intraspecific variation between channel catfish (*I. punctatus*) and Ictalurid hybrid catfish (*I. punctatus* x *I. furcatus*).

**Figure 1:**
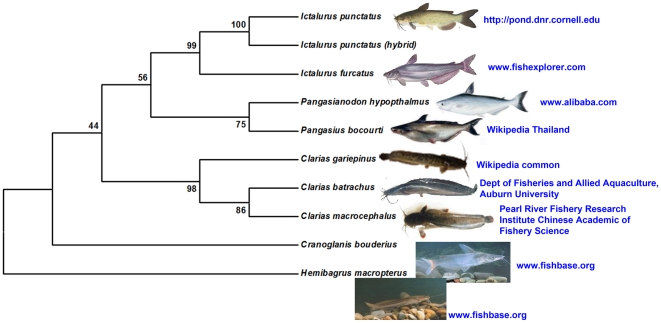
Phylogenetic consensus tree of nine catfish species (and an Ictalurid hybrid) constructed using Maximum Parsimony (MP) Method. The percentage of replicate trees in which the associated taxa clustered together in the bootstrap test (1000 replicates) is shown next to the branches [21]. *Hemibagrus macropterus* was used as an outgroup. The MP tree was obtained using the Close-Neighbor-Interchange algorithm [20] with search level 1 in which the initial trees were obtained with the random addition of sequences (10 replicates). The source for each image was displayed next to the pictures.

According to the Maximum Parsimony (MP) tree (Figure 1), the species in the present study were clustered independently within their corresponding genera. Three distinct subclades which consist of families Ictaluridae (*Ictalurus*, 2 species and a hybrid), Pangasiidae (*Pangasius* and *Pangasianodon*, 2 species) and Clariidae (*Clarias*, 3 species) were identified; supported by bootstrap values of 99%, 75% and 98% respectively. As presumed, *I. punctatus* and hybrid catfish (*I. punctatus* x *I. furcatus*) formed a cohesive group with a bootstrap value of 100%. Similarly, *C. batrachus* and *C. macrocephalus* created a subclade which was recognized with a moderately significant boostrap proportion of 0.86. Interestingly, Asian catfish represented by family Pangasiidae did not form an assemblage with another Asian catfish family Clariidae, but was found to be clustered together with family Ictaluridae before merging with Clariidae at a 44% bootstrap value. *H. macropterus* and *C. bouderius* appeared structured as individual subclades away from the other monophlyletic clades.

**Figure 2:**
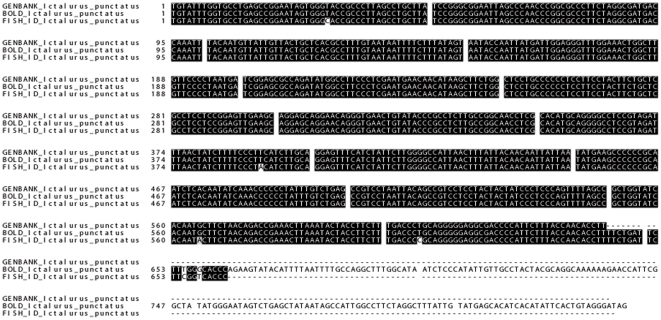
Multiple sequence alignment of consensus sequences for *Ictalurus punctatus* against voucher and reference sequences from GenBank and BOLD databases. A multiple sequence alignment of *Ictalurus punctatus* was generated by ClustalW and graphically represented by BOXSHADE 3.21. The nomenclature of the aligned sequences is as follows: FISH_ID_Ictalurus_punctatus (as *I. punctatus* consensus sequence in the present study), GENBANK_Ictalurus_punctatus (as GenBank voucher species consensus sequence) and BOLD_Ictalurus_punctatus (as BOLD species reference consensus sequence). Both voucher and reference sequences were downloaded from the two databases with the accession numbers listed in Table S2. Highly conserved regions which were ≥50% identical were boxed in solid black and light shading indicates conservative substitutions.

With the exception of poorly documented or mis-documented catfish species in GenBank and BOLD databases, multiple sequence alignments between consensus sequences (generated from this study) and consensus sequences from the two databases showed high identities (Figure 2). While small variations were observed among fish sequenced within a given species (Table 1), species-specific identifying sequences could be obtained in every case, usually with high concordance with existing database entries. All sequences from vouchered specimens used in the study were submitted to GenBank's Barcode database with accession numbers **JF292297-JF292429**. These sequences are also searchable through cross-referencing in the BOLD database. Additionally, all sequences (including consensus) generated in this study were used to create a searchable database as part of the larger catfish genome database (cBARBEL). The database can be found at http://www.animalgenome.org/catfish/fishid/. Users can search a barcode of interest again through one or all of the indexed species. The database will be updated as additional sequences and species are added.

## Discussion

### Species Identification Based on BLAST and BOLD

Regulatory scrutiny of seafood products and their labeling has lagged behind a surge in availability of imported wild-caught and aquaculture species in the United States. The particularly dramatic growth in catfish imports, their impact on the domestic catfish industry, and widespread questions regarding transparency in imported catfish origins and culture conditions, have combined to place catfish at the fore of emerging efforts to heighten fish product inspections in the U.S. A critical component of seafood inspections is determination of accuracy in species labeling. Molecular species identification using DNA barcoding has been applied successfully elsewhere but techniques and consensus barcodes had not been developed and validated in commercial catfish species. In this study, we have sequenced the COI region of the mitochondrial DNA to create a set of barcode sequences used to identify nine catfish species (and an Ictalurid hybrid) from five genera. We extensively compared our results to BOLD and GenBank databases records and found that, out of nine species studied, only five of them matched the reference sequences in both databases. The remaining species that were not perfectly aligned with the two databases included the three Clariid species listed in Table 2 and *H. macropterus*. Both *C. macrocephalus* and *H. macropterus* were yet to be barcoded in BOLD database, whereas *C. gariepinus* lacked any record in the Genbank database. On the other hand, our results brought into question the validity of *C. batrachus* in both databases.

Using our *C. batrachus* sequences as queries against the BOLD-IDS returned “no match.” BOLD-IDS validates its identification search only if the species in the reference database has at least three barcoded specimens and identifies the query sequences if it matches the reference sequence within the conspecific distance of less than 2% [19] or not exceeding 3% as suggested by Wong and Hanner [22]. Low (89%) matches were also recorded with *C. batrachus* sequences in GenBank. However, after re-examining the identification and sampling history of these specimens, we strongly suspected that the aberrant results revealed either that the *C. batrachus* sequences stored in both the BOLD and GenBank databases were originally specimens of *C. macrocephalus* or hybridized species that have been accidentally utilized in cataloging the barcodes. Therefore, correct species labeling, morphological taxonomy and voucher documentation should be prioritized in case that reassessment of spurious data is necessary [23]. Mislabeling is not unexpected since both of these species are genetically homologous [24] and morphologically similar. It has been reported that *C. macrocephalus* could not be distinguished from a female *C. batrachus* [25]. Furthermore, artificial hybridization of *C. macrocephalus* x *C. batrachus* for aquaculture purposes is increasingly popular [26]–[27]. Another possible explanation of low or unmatching results for *C. batrachus* specimens is that geographically divergent populations of *C. batrachus* may exist. This has been demonstrated in a karyological study which showed that *C. batrachus* from South Asia is distinctive from populations from Southeast Asia [28]–[30]. Therefore, some of the *C. batrachus* specimens barcoded in both databases may represent a subspecies from South Asia.

We encountered several difficulties in ascertaining the accuracy of BOLD and GenBank records that illustrate current shortcomings in these systems. BOLD data records and sequences often lack transparency for all but the most common species. For example, only one reference sequence for *C. macrocephalus* and C. *batrachus* is available for public viewing, despite more being deposited in the database. Lack of access to these additional sequences makes it hard to ascertain how species determinations are being made using the BOLD database.

Additionally, as mentioned above, a large percentage of publicly available barcodes in BOLD-IDS come from GenBank where there is high probability of tentative, incorrect or low-quality sequences being archived in an era of high-throughput sequencing. Additionally, the accuracy of sequence data cannot be verified given that sequence tracefiles or voucher samples are not retrievable via GenBank. Likewise, difficulties also arise in BOLD database to corroborate suspected records although greater effort is made on quality control [22]. For species with few records, mistakes in private submissions and/or records gleaned from GenBank can result in incorrect identification of samples sequences using the BOLD-IDS. Continual changes to private records and addition and subtraction of sequences can also change identification results obtained over time. Caution and due diligence is therefore required from the user seeking to utilize existing databases for barcode-based species identification.

### Sequence Divergence and Phylogenetic Analysis

One crucial barcoding criteria is that congeneric divergence should be higher than conspecific divergence [31]. While the sequence variation between five genera observed in this study was atypically high, averaging 18.3%, other studies showed a lower congeneric variance such as 7.48% in shark and rays [32], 8.37% in Canadian freshwater fishes [31], and 9.93% in Australian marine fishes [23]. In view of this, population genetic and taxonomic analysis will be able to provide a clearer picture of the evolutionary history of catfish in this study. A maximum genetic distance of 3% is sufficient to distinguish all the catfish in this study. As expected, species from the same genera were clustered tightly into a single clade with well supported bootstrap proportion [11]. Hybrid catfish with a maternal parent from *I. punctatus*, showed the expected result of barcoding as *I. punctatus* with minimal genetic distance (0.8%) resulting from intraspecific variation within channel catfish.

From Figure 1, Pangasiidae was observed as sister group to Ictaluridae albeit at a relatively low bootstrap percentage of 56%, whereas Bagridae represented by *H. macropterus* was the most diverged family from the rest of the groups [33]. Congruent with our data, Funk and Omland [34] has also found that the clustering of *C. macrocephalus* and *C. batrachus* in one lineage and *C. gariepinus* in another lineage resulted from their geographical separation during early stages of their evolution; with the former two species being native Asian catfish and the latter of African origin. The mean genetic distance between these two lineages is 14.7% (Table 4).

In conclusion, DNA barcoding is emerging as an invaluable tool to regulatory agencies and fisheries managers for species authentication, food safety, conservation management as well as consumer health and support [35]. Here, we have developed and validated DNA barcoding techniques and consensus sequences for important aquaculture and wild species of catfish. Our results indicate that DNA barcoding is a powerful technique, accurately identifying samples regardless of sample source. The barcodes have been deposited in a searchable catfish barcoding database that will be updated as additional samples and species are sequenced. The developed barcodes will aid in upcoming efforts to heighten U.S. fish products inspection and regulation requirements by ensuring accurate labeling of frozen and processed catfish products. Consensus barcodes from these species will also speed the development of fast-turnaround/high-throughput array or SNP-based assays based on informative COI polymorphic sites.

## Supporting Information

Table S1 Primers used for PCR amplification and sequencing.
(DOC)

Table S2 Reference sequence numbers (BOLD) and accession numbers (GenBank) of voucher species used to build multiple sequence alignment of *Ictalurus punctatus* using CLUSTALW program in Figure 2.
(DOC)
