# Supplementary material for: DNA Barcoding of Catfish: Species Authentication and Phylogenetic Assessment
Source: PLoS One. 2011 Mar 15;6(3):e17812. doi: 10.1371/journal.pone.0017812 (PMC3057997; doi:10.1371/journal.pone.0017812)
Supplement: Table S1 — Primers used for PCR amplification and sequencing. (DOC) [file pone.0017812.s001.doc]

**Table S1: Primers used for PCR amplification and sequencing**

| **Name** | **Primer Sequence (5'-3')** | **Reference** |
| --- | --- | --- |
| **C_FishF1t1** |  |  |
| VF2_t1 | 5’TGTAAAACGACGGCCAGTCAACCAACCACAAAGACATTGGCAC3' | Ward et al. 2005 |
| FishF2_t1 | 5’TGTAAAACGACGGCCAGTCGACTAATCATAAAGATATCGGCAC3' | Ward et al. 2005 |
| **C_FishR1t1** |  |  |
| FishR2_t1 | 5’CAGGAAACAGCTATGACACTTCAGGGTGACCGAAGAATCAGAA3' | Ward et al. 2005 |
| FR1d_t1 | 5’CAGGAAACAGCTATGACACCTCAGGGTGTCCGAARAAYCARAA3’ | Ivanova et al. 2007 |
| **M13F** | 5’TGTAAAACGACGGCCAGT3' | Messing 1983 |
| **M13R** | 5’CAGGAAACAGCTATGAC3’ | Messing 1983 |

**Cited References**

1. Ward RD, Zemlak TS, Innes BH, Last PR, Hebert PD (2005) DNA barcoding Australia's fish species. Philos Trans R Soc Lond B Biol Sci 360: 1847-1857.
2. Ivanova NV, Zemlak TS, Hanner RH, Hebert PDN (2007) Universal primers cocktails for fish DNA barcoding. Molecular Ecology Notes doi: 10.1111/j.1471-8286.2007.01748.x.
3. Messing J (1983) New M13 vectors for cloning. Methods Enzymol 101: 20-78.
