# Supplementary material for: DNA Barcoding of Catfish: Species Authentication and Phylogenetic Assessment
Source: PLoS One. 2011 Mar 15;6(3):e17812. doi: 10.1371/journal.pone.0017812 (PMC3057997; doi:10.1371/journal.pone.0017812)
Supplement: Table S2 — Reference sequence numbers (BOLD) and accession numbers (GenBank) of voucher species used to build multiple sequence alignment of Ictalurus punctatus using CLUSTALW program in Figure 2. (DOC) [file pone.0017812.s002.doc]

**Table S2 Reference sequence numbers (BOLD) and accession numbers (GenBank) of voucher species used to build multiple sequence alignment of *Ictalurus punctatus* using CLUSTALW program in Figure 2.**

| **No** | **BOLD Reference Sequences:** | **GenBank Accession Number:** |
| --- | --- | --- |
| 1 | GBGC0208-06|AF482987 | EU752103.1 |
| 2 | GBGC1573-06|NC_003489 | EU752102.1 |
| 3 | GBGC3968-07|AF482987 AF032380 AF032381 | EU752101.1 |
| 4 | GBGC6278-08|AF482987 AF032380 AF032381 | EU752100.1 |
| 5 | BCFB138-06|BCF-0501-1 | EU490865.1 |
| 6 | BCFB139-06|BCF-0501-2 | EU524686.1 |
| 7 | BCFB129-06|BCF-0114-1 | EU524684.1 |
| 8 | BCFB130-06|BCF-0114-2 | EU524683.1 |
| 9 | BCFB131-06|BCF-0114-3 | EU524682.1 |
| 10 | NHFEC062-06|BCF-0113-1 | EU524681.1 |
| 11 | BCFB132-06|BCF-0115-1 | EU524680.1 |
| 12 | BCFB133-06|BCF-0115-2 | EU524679.1 |
| 13 | BCFB134-06|BCF-0115-3 | EU524678.1 |
| 14 | BCFB135-06|BCF-0394-1 | EU524677.1 |
| 15 | BCFB136-06|BCF-0394-2 | EU524676.1 |
| 16 | BCFB137-06|BCF-0394-3 | EU524106.1 |
| 17 | GBGC3998-08|EU490865 |  |
| 18 | GBGC8082-09|AF482987 AF032380 AF032381 |  |
